# Geographic information system (GIS) maps and malaria control monitoring: intervention coverage and health outcome in distal villages of Khammouane province, Laos

**DOI:** 10.1186/1475-2875-8-217

**Published:** 2009-09-22

**Authors:** Yoshihisa Shirayama, Samlane Phompida, Kenji Shibuya

**Affiliations:** 1 Department of Global Health Policy, Graduate School of Medicine, University of Tokyo, Japan; 2 Center of Malariology, Parasitology & Entomology, Ministry of Health, Laos

## Abstract

**Background:**

Insecticide-treated nets (ITNs) are a key intervention to control malaria. The intervention coverage varies as a consequence of geographical accessibility to remote villages and limitations of financial and human resources for the intervention. People's adherence to the intervention, i.e., proper use of ITNs, also affects malaria health outcome. The study objective is to explore the impact of the intervention coverage and people's adherence to the intervention on malaria health outcome among targeted villages in various geographic locations.

**Methods:**

Geographic information system (GIS) maps were developed using the data collected in an active case detection survey in Khammouane province, Laos. The survey was conducted using rapid diagnostic tests (RDTs) and a structured questionnaire at 23 sites in the province from June to July, the rainy season, in 2005. A total of 1,711 villagers from 403 households participated in the survey.

**Results:**

As indicated on the GIS maps, villages with malaria cases, lower intervention coverage, and lower adherence were identified. Although no malaria case was detected in most villages with the best access to the district center, several cases were detected in the distal villages, where the intervention coverage and adherence to the intervention remained relatively lower.

**Conclusion:**

Based on the data and maps, it was demonstrated that malaria remained unevenly distributed within districts. Balancing the intervention coverage in the distal villages with the overall coverage and continued promotion of the proper use of ITNs are necessary for a further reduction of malaria cases in the province.

## Background

Insecticide-treated nets (ITNs) are a key intervention to control malaria [1,2]. One hundred percent coverage is ideal, however, in actuality, the intervention coverage varies as a consequence of geographical accessibility to the targeted villages and limitations of financial and human resources for the intervention. In operating the intervention, we need to make sure that: 1) people own enough ITNs for all family members, 2) sleep under ITNs every night, and 3) re-treat ITNs regularly. In addition to the availability of ITNs, the adherence to the intervention, i.e., proper use of ITNs, also affects malaria health outcome [3]. Through community health education, people learn about the cause of malaria and the proper use of ITNs, and are encouraged to visit the health facility for proper diagnosis and treatment rather than self-treatment.

In order to explore the impact of intervention coverage and people's adherence to the intervention on malaria health outcome among targeted villages in various geographic locations, geographic information system (GIS) maps were developed based on the data collected in a community-based, active case detection survey in Khammouane province, Laos. Detailed information about this survey was previously reported [4]. The survey was conducted using rapid diagnostic tests (RDTs) and a structured questionnaire at 23 sites in the province from June to July, the rainy season, in 2005. A total of 1,711 villagers from 403 households participated in the survey.

A growing number of GIS studies as well as review articles [5-9] indicate that GIS is a powerful tool for monitoring public health in various geographical locations. In the field of malaria control, GIS was mainly used for describing malaria risk, often limited to hospital-based morbidity and mortality. The efforts [10,11] to describe malaria risk continued expanding, and the Malaria Atlas Project (MAP) realized the first global endemicity map for *Plasmodium falciparum *based on 7,953 parasite rate surveys [12], including the surveys from Laos [13]. Upon request, the survey data in Khammouane province was also incorporated in this global project. At the same time, for practical use, more detailed maps with comprehensive information are in demand [14]. Such dynamic maps based on recent community-based data could be enormously valuable as an operational tool for planning actions against the described malaria risk.

Sipe and Pat reported that GIS for malaria control was most often used in sub-Saharan Africa and rarely used in Southeast Asia, and described challenges to its successful use, based on their literature reviews and their experience in Indonesia [15]. The challenges included inadequate data, financial cost of global positioning system (GPS) hardware devices and GIS software, and lack of technical specialists to give training to the field staff or to perform the analysis. Recently, GPS hardware devices have become inexpensive, easy to use, and available for personal use. User-friendly GIS software programs have been developed and some programs are provided free-of-charge. Although sophisticated GIS analysis would still be in the hands of specialists, GIS mapping technique, as a part of a series of GIS analyses, can be a helpful tool for any field workers who need to visualize and analyze the collected data immediately.

Barat reported that data-driven decision-making was one of the essential factors commonly observed in four countries where malaria burden was successfully reduced [16]. There is also an estimation that approximately 80% of the information needs of the local government decision-makers relate to geographical locations [17]. When feeding back the results of a field survey to the decision-makers and local health workers, visually-indicated GIS maps could be much more effective for communicating the main findings, rather than tables of statistical results alone. This paper shows how the data of a community-based survey was GIS-mapped and how these maps were used to identify where malaria control activity could be strengthened.

The objective of this study is to explore the impact of the intervention coverage and the adherence to the intervention on malaria health outcome, through the development of GIS maps based on the data collected in a community-based survey in Khammouane province, Laos.

## Methods

### Characteristics of study sites

Khammouane province is located about 350 km south-east of Vientiane, the capital of Laos. It has a tropical climate, an average temperature range of 30-34°C, average humidity of 60-80% and an annual rainfall of 500-600 mm [18]. Among nine districts in the province, three (Nyumarat, Bourapar, and Xaybotom) are at higher risk of malaria. The villages in these districts are populated predominantly by rice farmers of two ethnic groups: Lao Lum (major ethnic group) and Lao Thung (minor ethnic group). An entomological survey showed that the main malaria vectors in the province are *Anopheles minimus, Anopheles dirus*, and *Anopheles nivipes *[19,20]. There is no report of their resistance status to insecticides from this study area [21].

### Study samples

A community-based survey was conducted at 23 sites (20 villages and 3 district hospitals) in the three districts. Through discussion with local health staff, study villages were stratified, randomly selected to include both Lao Lum and Lao Thung villages at varying distances from the district hospital. The study team visited all the households in each village and studied all family members available. The study team visited 403 households in total and was ultimately able to interview 1,711 out of 2,409 family members (71.0%) in those households.

### Data collection

The researcher (Y. Shirayama), the medical doctors from the Lao Ministry of Health Center of Malariology, Parasitology and Entomology (CMPE), and local health staff conducted questionnaire interviews and malaria RDTs from June to July (during the rainy season) in 2005.

### GPS locations of the villages and health facilities

GPS is a satellite-based navigation system made up of a network of 24 satellites placed into orbit by the U.S. Department of Defense, officially called NAVSTAR satellite system. The interception of a minimum of three satellite signals allows the GPS receiver to calculate its position on the earth with respect to latitude and longitude. A minimum of four satellite signals is required to include altitude calculations. GPS receivers are accurate to within 15 meters on average. Garmin Geko™ 201, a GPS handheld receiver used in this study has a special system called *Wide Area Augmentation System (WAAS) *which can improve accuracy to less than three meters on average [22]. GPS locations (latitude, longitude, altitude) of the health facilities and the study villages were registered as *Waypoints*, and the study team's path of travel, called a *Track*, was also recorded throughout the field survey. When conducting a survey in rural areas without accurate maps available, nor roads, GPS is particularly useful.

### Components of GIS

**Figure 1:**
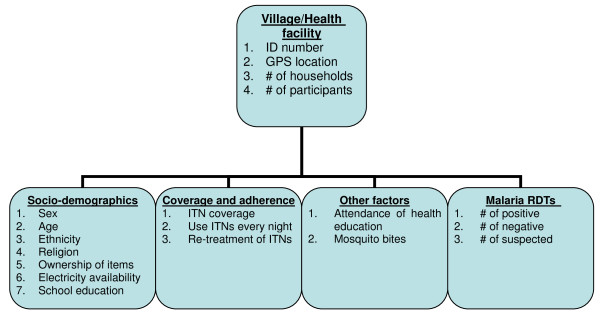
**Database components**.

Figure 1 shows the components of GIS of this study. The data were collected through questionnaire interviews and malaria RDTs at each household.

### Questionnaire interview results

Each family member of study households was interviewed using a structured questionnaire. The questionnaire consisted of questions relating to socio-demographic characteristics, prevention and treatment of malaria, contact with mosquitoes, community health education attendance, and knowledge of the cause of malaria.

### Definition of malaria health outcome

The *Paracheck *immunochromatographic test was used (Orchid biomedical system, Goa, India). The test detects the presence of *P. falciparum-*specific histidine-rich protein-2 (HRP-2) in a finger-prick blood sample and considered to be the most appropriate test kit [23-25]. The test was performed by medical doctors and local health staff according to the manufacturer's instructions.

### Development of GIS maps

GPS data was downloaded from the GPS handheld receiver. For GIS-mapping and analysis, *KASHMIR 3D Version 8.0.9 Beta *[26], and *Mandara for Windows Version 9.10 *were used [27]. Both software programmes are available free-of-charge on their respective websites, limited to the purpose of academic or non-commercial use. All GIS maps were converted into '.html' format so that GIS maps can be accessed using a standard internet browser without need for a specific viewing programme. Each variable was outputted into the map only in percentage, the number of each factor of interest divided by the total number of observations in each village.

### Study ethics

Approval for this study was obtained from the Lao Ministry of Health and the Ethics Committee of the Graduate School of Medicine, University of Tokyo, Tokyo, Japan (reference no. 1129, dated 23 March 2005). Written informed consent was obtained from each participant after the objectives of the study were explained. Free treatment was offered to patients diagnosed with malaria during the field survey.

## Results

On the developed maps, the provincial hospital and three district hospitals are shown as red cross icons, and blue lines are the *Track*, the study team's path of travel. Height above sea level ranged from 157.3 m to 546.6 m.

### Socio-demographic characteristics

**Figure 2:**
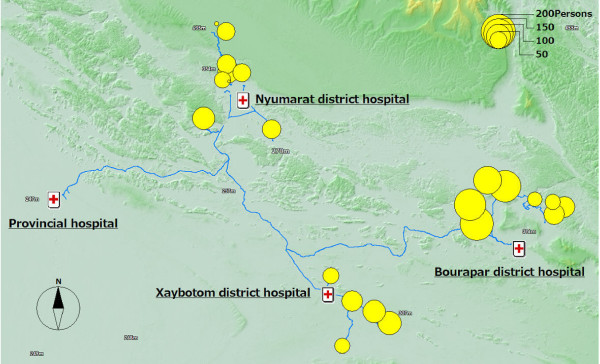
**The number of the study participants in each site**.

Figure 2 shows the number of study participants at each study site. Other maps are not presented in this paper, but each socio-demographic characteristic, i.e., sex, age, ethnicity, religion, income, ownership of a vehicle, TV, and radio, availability of electricity, and school education, can be outputted to a map. Out of the 1711 study participants, 46.5% were male and 22.0% were children under five. 68.8% were Lao Lum major ethnic group and 52.8% were Animist.

### Malaria health outcome

**Figure 3:**
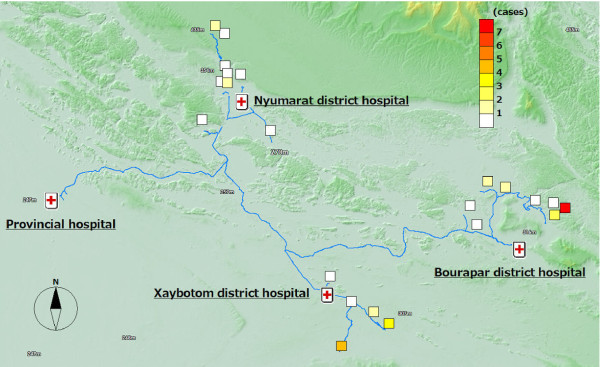
**Malaria health outcome: 21 positive and suspected cases**.

Figure 3 shows malaria health outcome. In total, 12 *P. falciparum *malaria positive cases were detected by RDTs. The proportion of positive cases was 0.7% (12/1,711) with a range in each village of 0~8.2%. Apart from these 12 positive cases, the medical doctors suspected that nine patients were *P. falciparum *malaria false-negative cases or were infected with other types of malaria, based on clinical signs such as fever, chills vomiting and headache. Those nine cases were also indicated on the maps.

### The intervention coverage and adherence to the intervention

**Figure 4:**
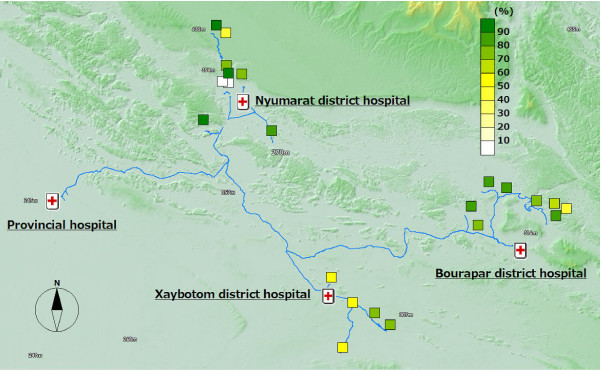
**ITN coverage**.

**Figure 5:**
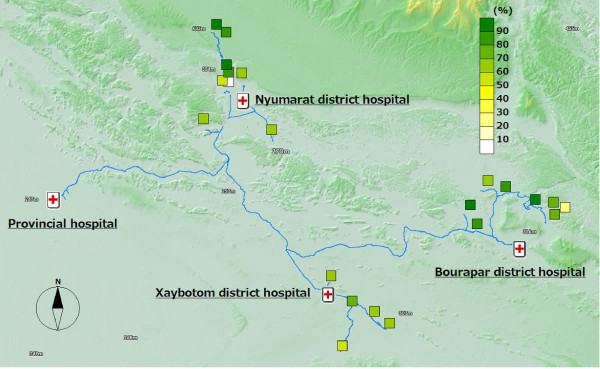
**Proportion of households where ITNs are re-treated annually**.

**Figure 6:**
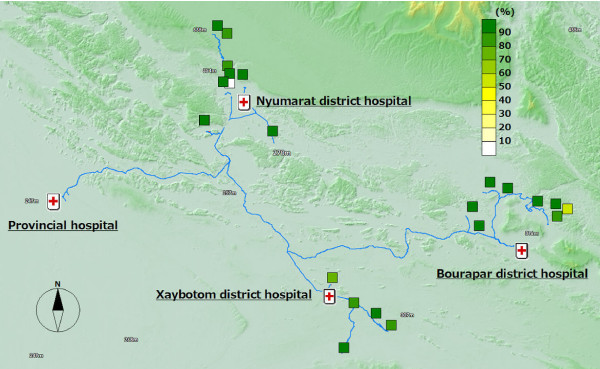
**Proportion of people who sleep under nets every night**.

**Figure 7:**
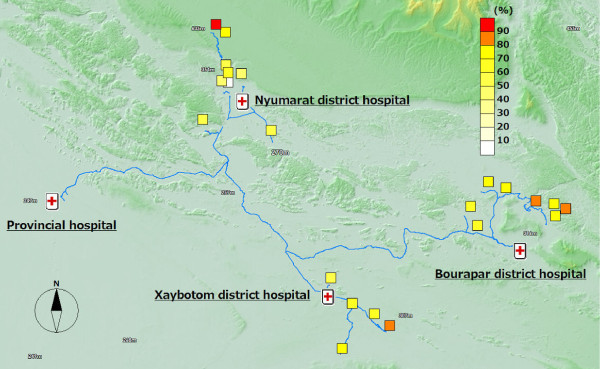
**Proportion of people who reported getting mosquito bites often**.

It is recommended that the number of persons who share the same ITN should be smaller than three in this project. In 76.7% (309/403) of households, the recommended intervention coverage was achieved (Figure 4), with a range in each village of 42.9-100%. In 73.9% (298/403) of households, ITNs were re-treated annually before the start of the rainy season (Figure 5) with a range in each village of 21.4-95.0%. 92.1% (1,575/1,711) reported that they slept in bed nets every night (Figure 6) with a range in each village of 53.4-98.9%. 87.3% (352/403) of household heads have attended a malaria education program at least once. The proportion of household heads who mentioned mosquito bites as the cause of malaria was 39.0% (157/403). 10.2% (174/1,711) visited the hospital for diagnosis and treatment of malaria. 57.6% (232/403) reported that they make sure to take all tablets of anti-malarial drugs as prescribed. To the question "How often do you get mosquito bites?" 68% (1,164/1,711) answered "Often" (Figure 7) with a range in each village of 49.1-83.3%.

### The coverage and health outcome in the distal (remote) villages

Out of the 12 malaria positive cases, almost all of them were detected in the distal villages. The villages with the best access to the district hospital, the central part of the district, were malaria-free. Six cases were detected from one remote village of Bourapar district. The risk of malaria infection in this village (8.2%, 6/73) was statistically significantly higher than that in other villages (P < 0.001, chi-square test). To travel to this remote village from the district hospital, it requires not only a road trip by car, but also three hours by boat on the flooded river during the rainy season. Notable characteristics specific to this village were longest travel time from the district hospital, low coverage of ITNs (Figure 4: 42.9% vs. total average 76.7%), low re-treatment rate (Figure 5: 21.4% vs. 73.9%), and low rate of bed net use every night (Figure 6: 53.4% vs. 92.1%).

In addition to the remote village with six positive cases, four more villages had malaria positive cases. These five villages with malaria cases were located in the area with least access from the central part of each district, and had lower re-treatment (Figure 5: 59.3% mean of proportions in the five villages vs. 76.4% mean of proportions in the other 15 villages) and higher proportion of people reporting frequent mosquito bites (Figure 7: 77.8% vs. 63.3%).

## Discussion

GIS maps visually indicated the uneven distribution of intervention coverage and health outcome within one province. They highlighted where malaria cases occurred, as well as villages with lower coverage of ITNs or lower adherence to the intervention. Based on the data and the maps, feedback was given to decision-makers and local health staff and helped with prioritizing where malaria control activity could be strengthened under limited financial and human resources.

In the distal villages located far from the district hospitals, malaria cases were detected, while the villages with the best access were malaria-free. Malaria control center staff stationed in the central provincial hospital and at each district hospital were not aware of the situation in those villages. When there were no more malaria patients at the hospital or in villages with easy access by car, they assumed an optimistic situation in the whole district and province. Through this community-based survey visiting every household in the villages, including ones in the most remote areas with poor access, and conducting a screening of malaria at the site, we were able to demonstrate that malaria did not vanish, but remained unevenly distributed within districts. Malaria control staff should be encouraged to access distal villages cut-off by poor infrastructure and improve the intervention and adherence coverage, even though it is a challenge especially during the rainy season.

Multiple indicator cluster surveys (MICS) 3, the third round of a nationally representative sample survey was carried out in Laos between March and June 2006 [28]. MICS 3 reported the percentage of households with at least one ITN as 51.6% in rural areas with road access and 41.6% in rural areas without road access. The percentage of children aged 0-59 months who slept under a bed net during the previous night was 89.1% in rural areas with road access and 79.0% in rural areas without road access. The percentage of children aged 0-59 months who were ill with fever in the last two weeks was 15.3% (343/2247) in rural areas with road access and 15.9% (190/1195) in rural areas without road access. There is a similar tendency in the nationally representative samples with these findings in Khammouane province: the intervention coverage and health outcome in the areas with comparatively less accessibility remain worse than in the villages with better access.

Donors such as the World Bank, the Asian Development Bank, the European Union, and Japan supported malaria control efforts in Laos. Global Fund has been the principal donor these days. There is increasing demand to study the current situation of malaria transmission after such control efforts, including the very remote areas where malaria persists and the situation remains largely unknown due to limited access. To adjust the malaria control strategies to the new malaria transmission pattern, and enhance the evidence base and accountability for donors, conducting more similar prevalence surveys will be useful.

The study does have some limitations. Questions such as "How often do you get mosquito bites?" were ambiguous and all the responses were self-reported, with no scale or criteria used to judge the validity. Regarding the precision of malaria diagnosis, it remains unknown exactly how many cases were false positive or false negative without the use of the gold standard microscopy method of detection. In this study, maps were created only at the village level, not at the individual household level. Many households are located adjacent to each other. It was technically difficult to capture exact GPS locations of homesteads in random formations, because a three to five meter square margin-of-error is the best GPS handheld receivers can provide. When conducting a survey in hard-to-reach, unmapped areas, maps at the village level could also provide valuable information to plan intervention targeting each village.

The maps were effective in communicating the main findings of the survey with the ministry staff and local health workers. When malaria control staff saw the maps, they realized their intervention coverage and health outcome achievements to date and gained confidence in their activities. Maps outputted as '.html' can be opened with a standard internet browser in computers available at the district hospital. Simply by clicking links in the browser's main window, local health staff can check distribution of each factor and refer to the original data. If we repeat the data collection or collect other variables such as entomological data in the future, this additional data can be integrated into the existing GIS maps. It is also possible that longitudinal data changing over space and time can be outputted as a series of GIS animation maps.

## Conclusion

GIS mapping allows visualization of field survey results, and provides essential information in targeting limited financial and human resources for the control of malaria within the province. The user-friendly GIS mapping method demonstrated in this study is a practical and feasible method for field researchers and health staff monitoring malaria risk in a geographically diverse area. The developed maps indicate the uneven distribution of intervention coverage and health outcome within the province. For a further reduction of malaria risk, balancing the intervention coverage in the distal villages with the overall coverage and continued promotion of the proper use of ITNs are necessary.

## Competing interests

The authors declare that they have no competing interests.

## Authors' contributions

YS designed the study, conducted the fieldwork, analyzed the data, and drafted the manuscript. SP participated in designing and coordinated the fieldwork. KS reviewed the manuscript and supervised implementation of the study. All authors read and approved the final manuscript.
